# Particular Aspects of Cardiac Rhythm Disorders in Pediatric Patients

**DOI:** 10.3390/children12020117

**Published:** 2025-01-22

**Authors:** Iuliana Moraru, Georgiana Bianca Constantin, Oana-Monica Duca, Iulia-Mariana Năstase, Raul Mihailov, Cristina Șerban, Mădălin Guliciuc

**Affiliations:** 1 Clinical Medical Department, Faculty of Medicine and Pharmacy, Dunarea de Jos University, 800216 Galati, Romania; iuliana.moraru@ugal.ro; 2 Clinical Emergency Hospital for Children Sf Ioan, 800487 Galati, Romania; 3 Morphological and Functional Sciences Department, Dunarea de Jos University, 800216 Galati, Romania; 4 Clinical Emergency County Hospital Braila, 810325 Braila, Romania; marwei91.iulia@gmail.com; 5 Clinical Surgical Department, Dunarea de Jos University, 800216 Galati, Romania; raul.mihailov@ugal.ro (R.M.); cristina.serban@ugal.ro (C.Ș.); madalin.guliciuc@ugal.ro (M.G.)

**Keywords:** cardiac rhythm disorders, children, sudden death

## Abstract

Background: Cardiac rhythm disorders in children and adolescents have a high incidence. In certain conditions, sudden death may occur. Materials and Methods: We conducted a prospective study including 199 patients divided into two subgroups: one subgroup of 80 practicing different sports and another one of 119 non-sportive, symptomatic patients. We studied all socio-demographic data, medical histories, and the elements revealed by the clinical and paraclinical examinations. Results: Only 39 (19.60%) participants did not practice any kind of sport; 46 participants had cases of sudden cardiac death in their families; 60.80% of children and adolescents were exposed to smoking; among the symptomatic group, symptoms like vertigo, chest pain, lipothymia, and palpitations predominated. Conclusions: A significant percentage of participants with heart rhythm disorders at risk for sudden death was identified. It is extremely important to identify and restrict them from intense physical exertion and to reassess them periodically in order to reduce the risk of sudden cardiac death.

## 1. Introduction

Asymptomatic or symptomatic heart rhythm disorders in the pediatric population and young people have a variable but rather high incidence. A small percentage of these can result in sudden death in certain situations, with physical exertion cited as a contributing factor.

Sudden cardiac death, a term that appeared in specialized literature in the 19th century, represents a clinical problem that has ventricular arrhythmias as its starting point. This fact has the direct consequence of the development of numerous pharmacological and non-pharmacological therapies, among which we mention transthoracic defibrillators, cardiac massage, and—in recent years—implantable defibrillators [1]. Currently, two hypotheses exist that underlie the molecular and ionic mechanisms of the physiological functionality of the heart. The first states that the so-called “voltage clock”, which has the predominant component of the “funny current” (If), contributes significantly to the potential of the stimulator [2]; the second hypothesis signals the importance of the “clock of Ca” in the proper functioning of the spontaneous process of diastolic depolarization [3].

Sudden cardiac death is a topic that has caused much controversy over time, especially in healthy children, young athletes, and those with pre-existing cardiac pathologies, with a general incidence of 8.6 per 100,000 in the population up to 40 years of age [4] and 1 in 50,000 to 1 in 80,000 athletes/year, frequently in males [5]. According to data from the specialized literature, sudden death in young athletes has a basis of not only primarily pre-existing structural heart defects (hypertrophic cardiomyopathy and congenital coronary anomalies) but also the presence of rhythm and conduction disorders. Young athletes seem to have a higher risk of sudden cardiac death due to acquired arrhythmogenic pathologies.

A study conducted in Italy identified arrhythmogenic right ventricular dysplasia as the main cause of sudden death in a group of young athletes [6]. Cardiac screening programs in the pediatric sports population are necessary for the timely identification of pre-existing or inherited cardiac pathologies, especially cardiac arrhythmias that can benefit from antiarrhythmic medication, thus reducing the risk of sudden death (SD). Most patients present minimal or even no symptoms, which makes it difficult to identify rhythm and conduction disorders in the absence of paraclinical means (electrocardiogram and Holter EKG monitoring).

In Romania, there are no studies to date regarding the incidence and particularities of cardiac rhythm disorders with the potential for SD in children and/or young athletes.

The aim of this study was to analyze the incidence of heart rhythm disorders in symptomatic children and apparently asymptomatic young athletes, both individually and comparing the two subgroups, correlating the diagnosis of arrhythmias with other sociodemographic and anamnestic factors, living conditions, and biological parameters.

The main objective of the study was to determine the overall incidence of heart rhythm disorders in the two subgroups, including arrhythmias that involve a risk of sudden death, and their correlations with possible prognostic factors.

## 2. Materials and Methods

We conducted an analytical, prospective study on 199 patients in the archives of the Emergency Clinical Hospital for Children “Sfântul Ioan” in Galati. The study was observational and longitudinal; the period in which it was carried out was 5 years between 2020 and 2024. For better collection and centralization of data, the observation sheets were revised, and personal follow-up sheets were drawn up for each individual patient in which their socio-demographic characteristics were noted, along with the necessary clinical and paraclinical data. The evaluation of these clinical and paraclinical parameters was performed either at the time of admission or at the time of presentation in the specialized outpatient clinic. The subsequent research was conducted after informed consent was obtained from the legal representatives of the patients, taking into account the current legislation of the World Health Organization and the European Union regarding research on human participants in the medical field as well as the latest version of the Helsinki Declaration of Human Rights. They were also informed about the possibility of receiving, upon the completion of the necessary tests, a medical contraindication consisting of the prohibition of physical exertion in the event that the patient presented rhythm disorders that may evolve later with the appearance of the risk of sudden death. The study was started after obtaining the approval of the Ethics Committee of the “Sf.Ioan” Galati Emergency Clinical Hospital (no 25222/9 October 2024).

The patients were divided into two subgroups:

a. Eighty pediatric patients who were practitioners of a performance sport that requires constant physical effort, followed over a period of 5 years, from January 2020 to January 2024.
b. The remaining 119 were pediatric, non-sportive, symptomatic patients. These patients were in the databases of the Emergency Clinical Hospital for children, “Sfântul Ioan” Galati, and were also followed for a period of 5 years, between January 2020 and January 2024.

This study was carried out in a phased manner. Thus, in the first stage, for centralization to be as correct as possible (from a medical point of view) and more coherent from an analytical point of view, we selected the patients who met the criteria to be part of this study. In the 2nd stage, the respondents were divided into two groups, depending on whether they were practicing a sport or not. In addition, the incidence of heart rhythm disorders was recorded, as well as the evolution of some pre-existing pathologies and associated comorbidities depending on the existence of certain demographic, socio-economic, cultural, and professional characteristics, as well as some clinical and paraclinical parameters that were of interest. In the 3rd stage, comprising an analysis of the information obtained from the corroboration of the data extracted from the patients’ observation sheets and the questionnaires given to the patients to fill in, comparisons were made between group A and B. The last stage of the study aimed to concretize an algorithm for the diagnosis, follow-up, and appropriate treatment of heart rhythm disorders present in the pediatric population and young people, symptomatic or athletes, with the aim of identifying and monitoring as accurately as possible the potentially lethal heart rhythm disorders.

From a clinical point of view, the following characteristics of pediatric patients, both athletes and non-athletes, were evaluated:

- Characteristics of perinatal evolution: type of birth, gestational age, APGAR score, birth weight, and association with perinatal pathologies;
- Current individual characteristics: current weight, waist, BMI, BP, and AV values (minimum, average, and maximum), the number of hours of sports performed per week, and consumption of stimulants or recommended chronic medication;
- Detecting the reasons for presentation at the time of the study;
- Corroboration of information to create the most complete anamnestic history: incidence, frequency, and severity of associated symptoms, presence of pathologic antecedents and family history of SD;
- Coexistence of some secondary pathologies in first-degree relatives: hypertension, cardiac disorders, diabetes, and metabolic syndrome.

In this study, we included pediatric patients, both athletes and non-athletes, aged between 1 and 18 years, while patients were excluded based on the following criteria:

- Their refusal to participate in the study;
- Existence of congenital cardiac malformations;
- Patients previously diagnosed with heart rhythm disorder;
- Heart surgeries;
- Other known neurological diseases or other disabilities.

### Statistical Analysis

The data resulted from the anamnesis and from the clinical and paraclinical examination of the patients, Holter ECG monitoring, and an exercise test, which were analyzed with the IBM SPSS Statistics 24 and Excel 2017 work programs; then, all the data were sorted according to the different criteria.

To obtain the graphical representations, we used pie, bar, and histogram graphs, and the statistical analyses were performed using software applications for the SPSS 24 and Excel 2017 programs. Initially, we calculated the raw descriptive statistical parameters for all variables for which this type of computational approach was potentially useful. These variables include the average value, the standard deviation, the minimum and maximum value (for continuous numerical variables), the frequency (for categorical variables), the value of the median and mode, skewness, and kurtosis indices. For categorical values, we used contingency tables and the non-parametric chi-square (χ^2^) test. That way, we made comparisons between frequencies by comparing the observed absolute frequencies with the theoretical ones, corresponding to the case where there was no association.

We also used descriptive statistics in order to calculate the central tendency and dispersion of the data (the 95% confidence interval), standard error of the mean, and the minimum and maximum value.

For continuous data, we analyzed simple bivariate correlations with the calculation of Pearson’s correlation coefficient (r—linear correlation coefficient) and determination factor (R^2^). Pearson’s correlation coefficient is useful for providing information on the direction and strength of the relationship for numerical variables.

Regarding the study of statistical significance, we calculated the “*p*” value, more specifically, the probability of accepting the null hypothesis—the situation when there is no association between the variables (meaning that to accept that there is an association, the value has to be less than a threshold of 5% or 1%).

We applied the Kendall-tau (rs) correlation test with two-tailed statistical significance testing for the categorical or ordinal data.

In order to highlight the statistically significant differences between the groups, the following statistical tests were also used: Student’s *t*-test and one-way ANOVA (for normally distributed continuous variables).

The results of the various analyses were considered statistically significant when an error probability value of *p* < 0.05 was obtained.

## 3. Results

The group of 199 patients was divided into subgroup A, that of asymptomatic athletes, representing 40.20% (n = 80), and subgroup B of symptomatic children, representing 59.80% (n = 119).

Regarding the living area, we observe a huge discrepancy between patients from urban areas and those from rural areas: 80.90% of the subjects were from an urban area and only 19.1% were from a rural area, a discrepancy which is present especially in subgroup A.

From the entire study group, a slightly higher incidence of females is noted, with a percentage of 55.20%, compared to males with a percentage of 44.72%.

Regarding eating habits, those who declared a balanced diet with healthy nutritional principles represent a higher percentage than those who consume junk food, at 64.82% versus 35.18%, respectively. However, given that we are talking about children and young athletes, we can consider the percentage of 35.18% who consume junk food to be worrying and significant. The chi-square test χ^2^ [χ^2^ = (70 − 55.5)·2/55.5 + (129 − 55.5)·2/55.5 = 101.12] had a value greater than the corresponding value of 3.8 from the Fisher table, so we can reject the null hypothesis and can conclude that the eating habits of the patients in the study group present a statistically significant disposition.

In terms of the APGAR score, we can see that none of the 199 subjects received an APGAR score of 10 at birth; however, 38.19% (n = 76) received a score of 9 and another 47.74% (n = 95) received a score of 8. It can be stated that a considerable percentage, 85.93% (n = 171), of those included in the study did not suffer during birth. Only 11.06% (n = 22) received an APGAR score of 7, and 3.02% (n = 6) were evaluated in the delivery room with a score of 6 (Table 1).

Regarding the evaluation of personal pathological antecedents that required hospitalization, we can conclude that, in the study group, a percentage of 13.57% had no admissions, the highest percentage, 41.21%, had two admissions, 23.12% had one admission, and 14.07% had three admissions, while a percentage of 8.04% had between four and ten admissions. From the point of view of the pathologies for which the subjects were hospitalized, respiratory and digestive pathologies predominate, as well as lipothymias (Table 1).

Regarding the information related to the health status of pediatric subjects according to indices such as the number of hours of sport performed weekly, the child’s current weight, height expressed in centimeters, blood sugar, and BMI, these data are grouped in Table 2.

Considering the incidence of sedentary lifestyles, compared to taking part in sports classes conducted in schools, we have the following data:

- N = 17 (8.54%) sedentary children compared to n = 182 (91.46%) active patients. Thus, the chi-square test χ^2^ [χ^2^ = (17 − 55.5)·2/55.5 + (182 − 55.5)·2/55.5 = 315.036] had a value greater than the corresponding value of 3.8 from the Fisher table, so we can reject the null hypothesis and can conclude that sedentary behavior presents a statistically significant disposition.
- At the same time, from the total of 199 pediatric patients, we can see that only 39 (19.60%) do not practice any kind of sport, not even the one provided in school programs, unlike the majority of the 160 subjects who do some performance sports in addition to sports from the school curriculum (80 performance athletes and 80 patients who are only enrolled in school sports). The chi-square test χ^2^ [χ^2^ = (39 − 55.5)·2/55.5 + (160 − 55.5)·2/55.5 = 201.66] had a value greater than the corresponding value of 3.8 from the Fisher table, so we can reject the null hypothesis and can conclude that practicing sports (even if it is just sports classes in schools) has a significant disposition in statistical terms.
- For the sports activity carried out in schools, we note the fact that there were 24 subjects (12.1%) who presented exemptions (either permanent—due to conditions that contraindicated physical effort—or occasional); for the remaining 175, there were no such prohibitions.
- With regard to the number of sports hours per week, they present extreme values (located between 0 and 15) and the associated distribution is not a normal Gaussian curve, but one deviated to the left. In this case, we can conclude that the majority of the subjects declared doing only two hours of sports per week (n = 95, 47.74%), while a minority declared six hours (n = 50, 25.13%).

**Figure 1:**
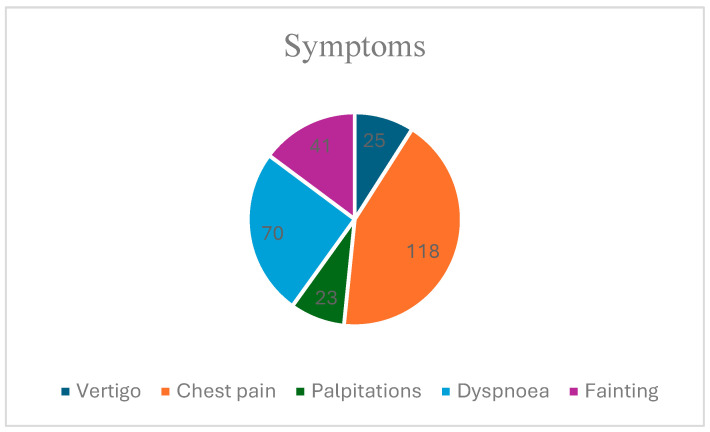
The distribution of symptoms.

Another element followed with particular attention among the 199 subjects of our study was represented by the presence of symptoms for which they went to the hospital or those declared according to the analysis sheets. We are therefore talking about the documentation of symptoms such as vertigo, chest pain, palpitations, dyspnea, and lipothymia (Figure 1). These clinical elements were later used to predict the occurrence of cardiac pathologies.

**Figure 2:**
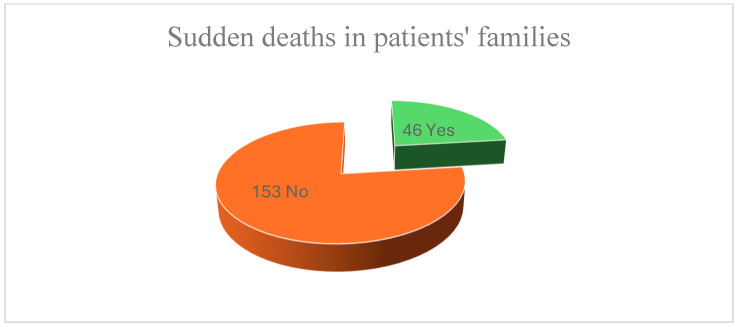
The distribution of family medical history.

In regard to the incidence of family history that may be responsible for increasing the risk of associating cardiac disorders, as we can see from Figure 2, 116 patients have a positive history. Carrying out an equation for the chi-square test gives us as a value of 79.37, an index higher than the reference number corresponding to the Fisher table, which proves that the presence of family history presents statistical significance.

On the other hand, in the case of the presence of sudden deaths, the weights are arranged in a distinct manner this time: 46 subjects mentioned having had cases of SD in the family (either in first-degree or in second-degree relatives) of cardiac origin, due to myocardial infarction, or which has as its starting point a neurological pathology, for example, stroke. The applicability of the chi-square test is possible in this case, giving us a value equal to 172.90. This is higher than the reference value in the Fisher table, which confirms that SD is a statistically significant index.

Regarding the number of hours that they spend daily in front of the laptop, TV, phones, or other gadgets, most subjects spend at least 2 h a day (n = 95, 47.74%), followed by those who allocate 3 h a day to such activities (n = 45), and finally, those who spend 4 h or more (n = 20). There is a direct, inversely proportional correlation between the number of hours that the subjects spend with electronic devices and the number of hours of physical activity.

Most patients deny stimulant use (n = 122) and chronic treatment (n = 97). In the case of the latter, it can be observed that the subjects mainly consume vitamins (31.16%), immunostimulants, anabolics, and calcium, both individually and in combinations. Among the most frequently used stimulants are coffee (27.14% of the subjects), closely followed by chocolate (6.53%), energizing agents, and the simultaneous administration of two or more products.

We observed a high exposure to passive smoking, with 60.80% of children and adolescents being exposed for an indefinite period of time. This raises a big question as to why such an alarming percentage of children under the age of 17 are exposed to such danger. The low education level of the parents is probably the most important factor. Even more alarming is the fact that 43.80% of the 121 patients are performance athletes. Moreover, 29 subjects (14.57%) are exposed to both passive and active smoking, a fact that indicates, for them, a greater risk of later developing other conditions.

The clinical evaluation revealed an extremely high percentage, 92.96% (n = 186), of children with no heart murmur; in 2.02% (n = 4), a grade I systolic murmur was identified, and in 5.03%, a grade II systolic murmur. No subject was identified as having grade III-VI murmurs. Identified murmurs were considered functional, and cardiac ultrasound was not performed in the study, as it would have been an element of paraclinical investigation that could not be applied to the entire group.

In total, 54.27% (n = 108 patients) did not present atrial extrasystoles (AEs) during Holter ECG monitoring. A significant percentage, 27.14% (n = 54), presented AEs but less than 1% of the number of recorded beats, and a percentage of 18.59% (n = 37) recorded AEs of more than 1% of the recorded Holter ECG beats. Of the entire group, 19.2% presented systematized atrial extrasystoles: 10.61% (n = 21) with bigeminism and 8.59% (n = 17) with trigeminism.

In total, 59.30% (n = 118) of the patients did not experience ventricular extrasystoles (VEs) during Holter monitoring. In total, 40.70% of subjects were identified as having VEs, of which 30.15% (n = 60) were below 1% of overall beats and 10.55% (n = 21) were above 1%. Among them, 28.64% (n = 57) presented systematization of ventricular extrasystoles.

In total, 71.36% (n = 142) were diagnosed with sinus tachycardia.

A small but significant percentage for the pathology, 9.05% (n = 18), recorded episodes of paroxysmal supraventricular tachycardia.

In total, 6.53% (n = 13) presented episodes of ventricular tachycardia.

No patient was recorded as having torsade de pointes.

The incidence of Wolff–Parkinson–White syndrome in the whole group was 20.6% (n = 41).

**Figure 3:**
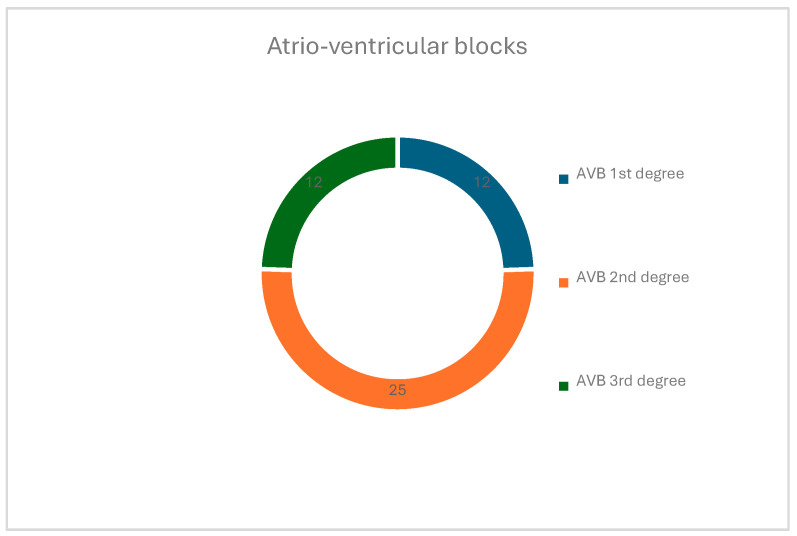
Distribution of the atrioventricular block types.

Regarding the blocks, 20.10% (n = 40) were recorded as having right bundle branch block and 12.56% (n = 25) as having left bundle branch block, alone or in association with other changes. Referring to the atrioventricular blocks, we can conclude that a percentage of 6.03% (n = 12) were identified as having first-degree atrioventricular block, 12.56% (n = 25) as having second-degree atrioventricular block, and another 6.03% (n = 12) as having complete block (Figure 3). It is extremely important to identify subjects with complete atrioventricular block (AVB), as well as monitoring and evaluating the need for implantation of a pacemaker device (a heart rate below 50 beats per minute in its absence), with the risk of SD being recognized. Relative to the total number of patients in the present study, the percentage of those with complete AVB is extremely alarming.

## 4. Discussion

Cardiac rhythm disorders in children and young people are considered common pathologies, in most cases being benign, but sometimes with a risk of sudden cardiac death. From a clinical point of view, they can be differentiated into two categories: asymptomatic and symptomatic; they are often diagnosed by chance during routine consultations. The data from the specialized literature show that those who are asymptomatic are at risk of SD. Symptomatic ones are mainly manifested by vertigo, palpitations, chest pains, dyspnea, and lipothymia [7]. Among these symptoms, it seems that those most frequently associated with malignant heart rhythm disorders are lipothymia and palpitations [8,9,10,11]. The history of sudden death in the family is also recognized in the literature as a risk factor [12,13].

We observed a large discrepancy between the patients from urban areas (80.90%) and those from rural areas (19.1%). Not only does the high percentage of patients correspond to those from urban areas (n = 161), but this is also the case for sports subjects (n = 79 sports patients from the urban area, compared to only 1 patient from the rural area). This underlines once again the extremely low and precarious addressability of healthcare in Romania for those from rural areas.

In our study group, the incidence of the female sex predominates, contrary to the data in the literature, which reveal a higher incidence of rhythm disorders in males [14,15,16]. Regarding the gender distribution in the whole group, a slightly higher percentage of female subjects is observed, 55.28%, compared to the male subjects included in the study, with a percentage of 44.72%. And with regard to the incidence of identified rhythm disorders, we can see that it is higher in the female sex in both analyzed subgroups.

One third of the total 199 subjects included in this study have unhealthy eating habits versus two-thirds who declared that they follow a balanced and healthy lifestyle.

It can be stated that the majority of the subjects included in the study did not suffer at birth. Only 11.06% (n = 22) received an APGAR score of 7, and 3.02% (n = 6) were assessed in the delivery room as having a score of 6.

The prevalence of patients complaining about palpitations in the whole group is 45.72% (n = 91), an extremely high percentage; among them, 11.55% (n = 23) mention these episodes as being occasional, while 34.17% (n = 68) declare them to be frequent. Palpitations are an extremely common pathology in pediatric practice; often episodes of palpitations occur during intense physical exertion, or in the context of fever or infectious diseases. Palpitations that start suddenly last only a few seconds or 1–2 min and stop just as they begin; they are very likely to be caused by an arrhythmia with a risk of sudden death [17]. Also, the patients whose episodes of palpitations are associated with lipothymia are at risk of SD according to sudden cardiac death studies [17,18,19,20,21].

In February 2019, Bobbo M. published a retrospective study (2009–2015) analyzing the files of patients who presented in the emergency department of an Italian hospital for palpitations. Bobbo concluded that the incidence of presentations for palpitations was only 0.07% of all presentations but points out that a very high percentage (13.5%) were diagnosed with a heart rhythm disorder with SD potential. Compared to previous studies where the predominant incidence was in the male sex, this time the female sex registers a slightly higher percentage at 52.1%. Also, more than half of the patients diagnosed with arrhythmia, 53.8%, received drug treatment, and 46.1% underwent ablation procedures for episodes of supraventricular tachycardia [22,23].

Chest pain is an alarming symptom for children and their relatives. A study published in 2001 reports that 98% of chest pains occurring in the pediatric and young population are not of cardiac origin [24]. Of all cases with chest pain as the reason for presentation, Selbst mentions a percentage of 6% as being of a cardiac nature. Of these, a very small percentage is secondary to cardiac arrhythmias [25]. In a study published in 2016, 27.7% of children identified as having VEs had chest pain as a symptom [26]. Therefore, although the incidence of chest pain in children and young people is very high, as is highlighted in our study where the percentage of those who declare episodes of chest pain is 59.29% (n = 118), only a small percentage are due to arrhythmias. In our group, 50.25% declare that these episodes are frequent, and only 9.04% are occasional.

Other symptoms of cardiac arrhythmias include vertigo. In the specialized literature, there are no percentages regarding the incidence of episodes of vertigo in heart rhythm disorders. Vertigo can be signaled in the case of supraventricular arrhythmias or in atrioventricular blocks. In the present study, 12.56% of the patients declared that they experienced episodes of vertigo, with all of them belonging to group B.

Almost three quarters of those monitored with Holter ECG were diagnosed with sinus tachycardia.

A small percentage, but significant for the pathology, recorded episodes of paroxysmal supraventricular tachycardia.

## 5. Conclusions

In the analyzed group, we found poor medical assistance, as well as low health education, in the rural area, which explains the very large discrepancy between those who come from urban areas versus those from rural areas. In subgroup A of athletes, only one patient was from a rural area.

Regarding the evaluation of personal pathological antecedents and the pathology that required hospitalization, we can conclude that most subjects had two hospitalizations each. From the point of view of the pathologies for which the subjects were hospitalized, respiratory and digestive pathologies predominate, as well as lipothymias.

Concerning the incidence of family history that may be responsible for increasing the risk of associating cardiac disorders, more than half of the subjects have a positive history. The chi-square test demonstrates that this is statistically significant.

The same thing is proved by performing the chi-square test also with regard to the history of sudden death, with about a quarter of the total number of subjects having a history of sudden death in their families.

The incidence of passive and active smoking in this study is alarmingly high. Statistically, it has been shown that there are correlations between both types of smoking and the identification of dangerous arrhythmias.

A significant percentage of subjects, both among athletes and symptomatic patients, with heart rhythm disorders at risk for SD have been identified. We consider it extremely important to identify and restrict them from intense physical exertion, to track and reassess them periodically, and thus to avoid the risk of sudden cardiac death.

### 5.1. Limitations of the Research

- Composing the group of athletes proved difficult; initially, we wanted this group to include 100 subjects. Many potential subjects refused to participate after being informed that there was a possibility that, following this study, they would be withdrawn from sports activities if they were identified as having a cardiac rhythm disorder at risk for SD;
- Compliance during monitoring of patients who were identified as having heart rhythm disorders at risk of SD was extremely low among athletes.

### 5.2. Future Research Perspectives

- Follow-ups should be conducted over the next 10–15 years with the subjects who were identified as having arrhythmias at risk of SD, both athletes and symptomatic, and the stratification of SD incidence among them accordingly.
- A study should be carried out on Wolff–Parkinson–White syndrome in the same socio-demographic area to identify the causes of such a high incidence of this syndrome, as well as an evaluation of the first-degree relatives of these subjects to establish correlations regarding the genetic component.

## Figures and Tables

**Table 1:** Distribution of patients according to age, birth weight, APGAR score, and number of admissions.

	Age (Years)	Weight at Birth (Grams)	APGAR Score	Number of Admissions
N Valid	199	199	199	199
Missing	0	0	0	0
Mean	12.03	3208.79	8.21	1.85
Median	13.00	3200.00	8.00	2.00
Std. Deviation	4.017	545.029	0.756	1.282
Skewness	−0.792	−0.595	−0.797	1.593
Std. Error of Skewness	0.172	0.172	0.172	0.172
Kurtosis	−0.190	0.273	0.468	7.886
Std. Error of Kurtosis	0.343	0.343	0.343	0.343
Minimum	1	1500	6	0
Maximum	18	4200	9	10

**Table 2:** Correlations of patients’ general health status.

	Number of Sport Hours/Week	Weight (kg)	Height (cm)	Body Mass Index (BMI)	Glycemia
N Valid	199	199	199	199	199
Missing	0	0	0	0	0
Mean	3.74	47.06	149.82	22.1413	91.58
Median	2.00	47.00	155.00	19.3625	90.00
Std. Deviation	3.186	20.432	23.741	36.37426	12.492
Skewness	1.244	1.017	−1.167	13.829	0.220
Std. Error of Skewness	0.172	0.172	0.172	0.172	0.172
Kurtosis	1.600	4.628	1.586	193.753	−0.154
Std. Error of Kurtosis	0.343	0.343	0.343	0.343	0.343
Minimum	0	7	56	11.11	65
Maximum	15	166	187	529.34	128

## Data Availability

The original contributions presented in this study are included in the article. Further inquiries can be directed to the corresponding author.
